# Electroless Gold-Modified Diatoms as Surface-Enhanced Raman Scattering Supports

**DOI:** 10.1186/s11671-016-1539-x

**Published:** 2016-06-29

**Authors:** Marianna Pannico, Ilaria Rea, Soundarrajan Chandrasekaran, Pellegrino Musto, Nicolas H. Voelcker, Luca De Stefano

**Affiliations:** Institute for Polymers, Composite and Biomaterials, Pozzuoli, NA Italy; Institute for Microelectronics and Microsystems, Via P. Castellino 131, Naples, 80131 Italy; Future Industries Institute, University of South Australia, Mawson Lakes Blvd, Adelaide, Australia

**Keywords:** SERS, Diatoms, p-Mercaptoaniline

## Abstract

Porous biosilica from diatom frustules is well known for its peculiar optical and mechanical properties. In this work, gold-coated diatom frustules are used as low-cost, ready available, functional support for surface-enhanced Raman scattering. Due to the morphology of the nanostructured surface and the smoothness of gold deposition via an electroless process, an enhancement factor for the p-mercaptoaniline Raman signal of the order of 10^5^ is obtained.

## Background

Surface-enhanced Raman scattering (SERS) is a powerful tool in the fields of analytical chemistry, surface science, electrochemistry, biology, and materials research [1–4]. Its high sensitivity and low detection limit (concentrations of <10^−8^ M), along with reports of single molecule detection, are key advantages that make SERS suitable even in situations where normal Raman spectroscopy fails: Raman scattering signal is usually very weak since its cross section is very low (from 10^3^ to 10^6^ times weaker than linear Rayleigh scattering); by coupling the electromagnetic field of the incoming probe laser light with the plasmonic field (i.e., surface charge density oscillations of the metal conduction electrons) of the metallic surface, the Raman scattering cross section is greatly enhanced, and therefore also the Raman signal. Moreover, the interaction between molecules under investigation and the metal support could introduce some specific features in the Raman spectrum registered, which can be used for analytical purposes. To obtain appreciable enhancement of Raman scattering, a noble metal surface of nanometer-scale roughness covering macroscopic dimensions area is necessary. Therefore, appropriately prepared substrates are essential to obtain SERS signal enhancement [5–10]. The best signal enhancement factors are currently obtained using silver nanostructured surfaces that unfortunately undergo a sudden oxidation during measurement, making their use as stable supports impractical. In order to overcome material limitations, during the last decade, new classes of artificial materials known as metamaterials and “photonic crystals” and “photonic quasi-crystals” have shown to be very effective SERS substrates, including for applications in biochemical sensing [11, 12]. The production of such optical structures requires a highly specialized design and sophisticated nanoelectronic fabrication techniques, particularly time consuming and costly, such as electron-beam lithography or nanoimprinting, which are not always available in small laboratories. More accessible and cheaper alternative approaches are therefore needed. Diatoms are a group of single-celled photosynthetic algae that possesses microscale shells made of hydrated amorphous silica (SiO_2_), called frustules [13]. Frustules show very ornate surface nanoscale pore patterns forming an amazing range of intricate designs with hierarchical ordered dimensions that span from nanoscale to microscale. Due to the quasi-ordered pore patterns, diatoms are endowed with peculiar optical properties including photonic properties, which have been extensively studied in the last few years [14–16]. Unexpected optical properties, due to pores spatial arrangement, such as diffraction-driven self-focusing [17], and gas-sensitive photoluminescence emission, related to material composition, have been demonstrated [18].

In this contribution, we have synthetized, by an innovative method based on electroless deposition of gold, metal-coated diatom frustules that can be exploited as effective supports for SERS.

## Methods

The Au plating solution (Oromerse Part B) was purchased from Technic, Inc. (USA). Tin(II) chloride, trifluoroacetic acid, ammonia, methanol, formaldehyde, sodium sulfite, sodium bicarbonate, sulfuric acid, and ethanol were supplied from Sigma-Aldrich (Australia). AgNO_3_ was obtained from Proscitech (Australia). p-Mercaptoaniline (pMA) 97 % was purchased from Sigma-Aldrich (Italy) and was used without further purification. Diatom frustules of *Aulacoseria* sp. were kindly furnished by Prof. D. Losic, University of Adelaide, Australia.

Diatom frustules were first incubated with hydrogen peroxide solution (35 %) over 24 h to increase the density of oxygen groups on the surface. The electroless Au deposition process includes three steps: sensitization of frustules in a solution of 0.026 M SnCl_2_ and 0.07 M trifluoroacetic acid for 45 min followed by rinsing in Milli-Q water; activation step, where the sensitized frustules were immersed into a 100-mL ammoniacal solution of 0.029 M AgNO_3_ for 30 min. During the activation step, a redox reaction helps in the deposition of Ag layer on the sensitized frustules. In the plating/galvanic displacement step, the activated frustules were immersed into a standard prebath solution for 1 h followed by a plating bath for the required deposition time (16–88 h). During this step, the Ag layer was galvanically displaced by Au in the plating bath solution which was kept at approximately 1 °C. The Au layer helps in the further oxidation of formaldehyde and concurrent reduction of Au(I) to Au(0), thus forming multiple layers of Au particles. The plating bath was prepared by mixing Au plating solution (Oromerse Part B, USA) containing 0.079 M Na_3_Au(SO_3_)_2_, 0.127 M Na_2_SO_3_, 0.625 M formaldehyde, and 0.025 M NaHCO_3_ in Milli-Q water. The pH was adjusted to 8–8.5 using sulphuric acid [19, 20].

Gold-coated frustules were examined by scanning electron microscopy (JEOL JSM-6400) equipped by energy dispersive X-ray analysis (EDAX) spectrometer (Noran Instruments Voyager Series IV).

The Raman spectra of pMA (both spontaneous and surface enhanced) were collected by a confocal Raman spectrometer (Horiba-Jobin Yvon Mod. Labspec Aramis) operating with a diode laser excitation source emitting at 785 nm. The 180° back-scattered radiation was collected by an Olympus metallurgical objective (MPlan 50x, NA = 0.75); a grating with 600 grooves/mm was used throughout. The radiation was focused onto a CCD detector (Synapse Mod. 354308) cooled at −70 °C by a Peltier module. Spectra were registered in the Raman-shift range 800–1800 cm^−1^. The laser power measured at the output of the objective was 18.7 mW, which resulted in about 17 mW/μm^2^ in terms of power density. The Raman band of a silicon wafer at 520 cm^−1^ was used to calibrate the spectrometer. Collection times were 3 s for non-SERS and 5 s for SERS spectra.

## Results and Discussion

**Fig. 1:**
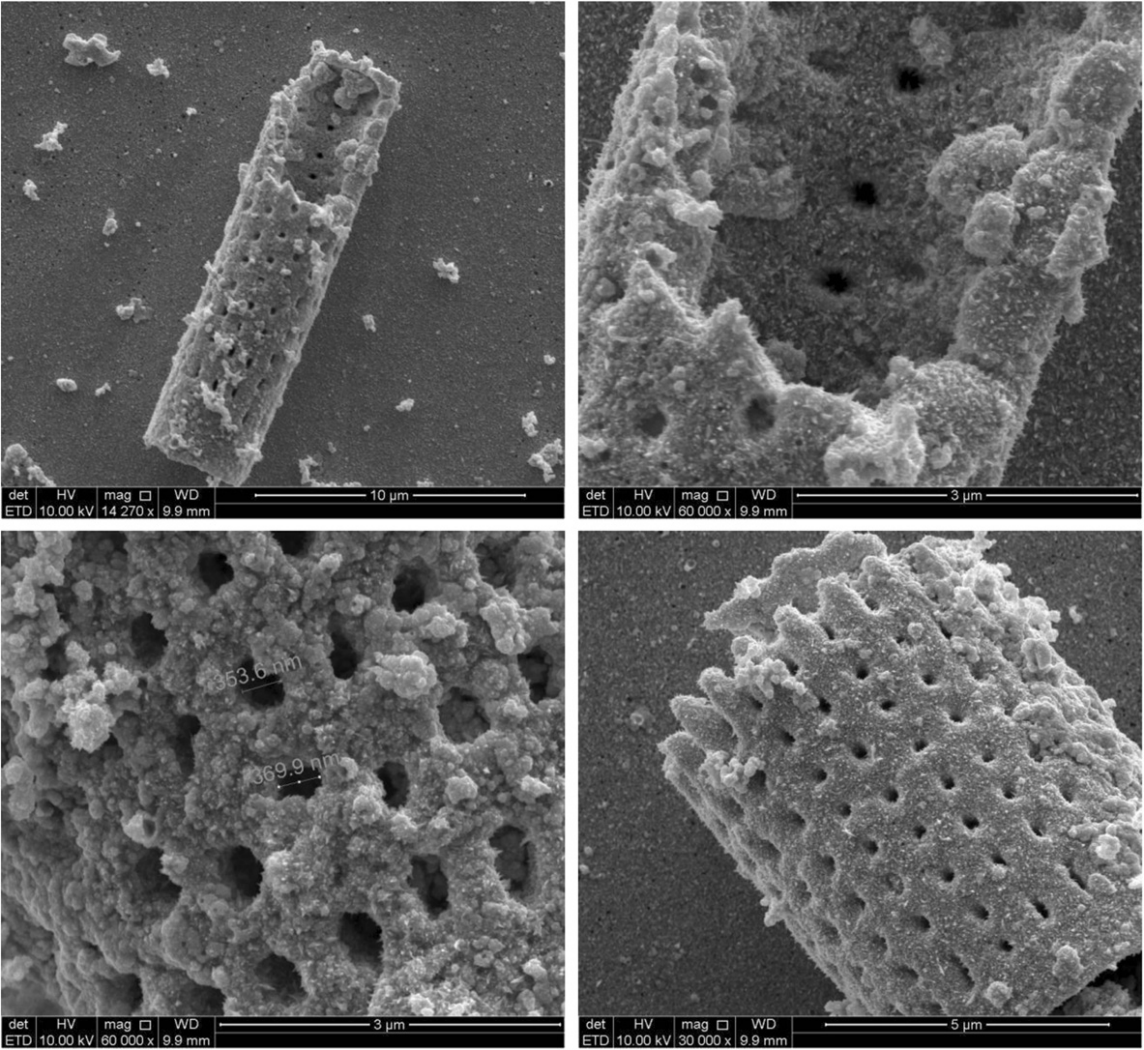
(**a**, *up left*) Single micrometric diatom frustule. (**b**, *up right*) Au nanoparticle covers homogeneously the outer and inner surfaces of the frustule. (**c**, *down left*) An enlargement of outer surface with pore size measurement. (**d**, *down right*) A smaller micrometric frustule. All images are relative to 48 h of Au deposition time

**Fig. 2:**
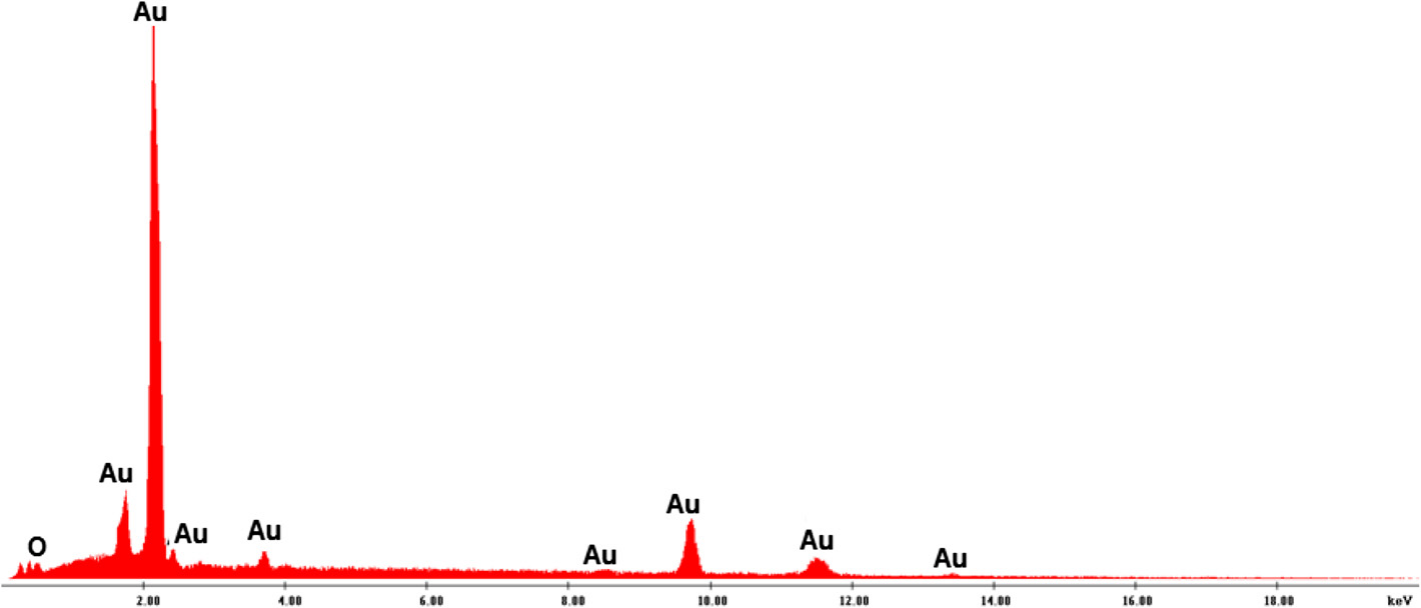
EDAX signal from diatom surface shows only Au characteristic peaks

**Fig. 3:**
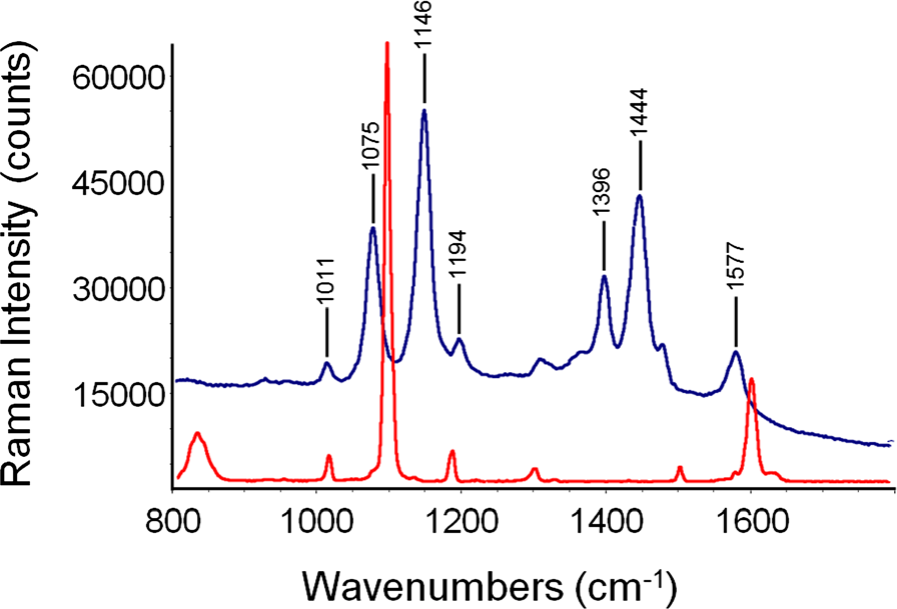
Raman (*red trace*) and SERS (*blue trace*) spectrum of pMA. The estimated number of sampled molecules is 2.55 × 1011 for the Raman spectrum and 2.90 × 106 for SERS, so that an enhancement factor of about 105 can be estimated

**Fig. 4:**
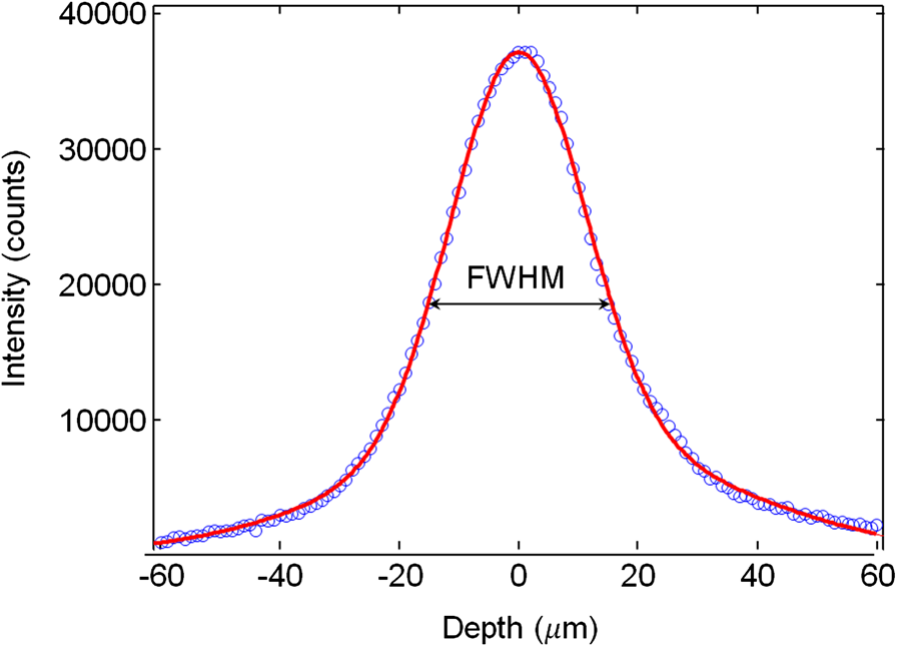
Depth profile on a silicon wafer to evaluate the sampling volume (Bν)

The key issue for high-performance SERS substrate is the existence of a uniform, continuous, thin layer of nanocrystalline metal onto the underlying 3D structure: the metal-dielectric interface is essential for both plasmon coupling and repeatable generation of SERS signal. Electroless deposition afforded a high density of Au nanoparticles on frustule surface that acted as preferred sites for further Au nucleation and growth during exposure to the plating solution [21]. The result was a complete coverage of the diatom frustules surface by a nanometric Au film, which completely preserved the original shape of the diatom micro-shell. Secondary electron (SE) images of *Aulacoseira* sp. diatom frustules after electroless deposition are reported in Fig. 1: the cylindrical-shaped frustules were micrometric in size (see Fig. 1a, d; 5–10 μm were total lengths of frustules) and showed ordered lines of nanometric pores (see Fig. 1b, c; 350–370 nm diameters); the outer and inner surfaces were covered by a fine grain Au layer (few nanometers of gold particles strongly interconnected) with some bigger coarse particle agglomerations. The EDAX spectra of the diatoms after electroless deposition confirmed the modified chemical nature of the surface since only the Au characteristic peaks were present in the spectrum reported in Fig. 2. The sensitivity of the SERS sensor based on Au-coated diatoms frustules (Au diatoms) was evaluated by measuring the scattering intensity of p-mercaptoaniline (pMA) probe molecules adsorbed on diatoms. Bonding of pMA to Au surfaces is generally believed to occur via a covalent interaction [22] with hydrogen loss from the thiol group and, therefore, is to be considered a chemisorption rather than a physisorption process. This reactivity produced compact, self-assembled monolayers (SAM) on the substrate surface [23–26]. Before carrying out SERS measurements, the Au-diatom powder was diluted in distilled water and dropped on an optical glass slide. After drying, a 10 μM pMA aqueous solution was dropped on the Au diatoms and dried at room temperature. In the confocal sampling mode, with the adopted objective, a spatial resolution of 1.7 μm^2^ is achieved in the *x-y* plane. Therefore, single diatoms can be spectroscopically characterized, collecting several spectra in different positions. The resulting SERS spectrum, in the range between 800 and 1800 cm^−1^ is shown in Fig. 3 (blue trace). For comparison, the spontaneous Raman spectrum of pMA powder (red trace in Fig. 3) is also reported. The intensities of the two spectra only seem to be comparable, since the number of molecules that generated the signals was very different. The number of molecules which contribute to the spontaneous Raman scattering (*N*_REF_ *=* 2.55 × 10^11^) was calculated as *N*_REF_ = (*B*_ν_ × *D*_pMA_/*M*_pMA_) × *A*, where *B*_ν_ is the optical excitation volume, *D*_pMA_ is the density of the pMA crystal at room temperature (1.06 g cm^3^), *A* is the Avogadro number and *M*_pMA_ is the pMA molecular weight (125.19 g/mol). *B*_*v*_ depends on the Raman operating parameters. It was estimated by carrying out *z* profile measurements on a silicon substrate under the same operating conditions used for the acquisitions. By varying the focus of the laser on the silicon sample (*z* profile), the intensity variation of the silicon peak (520 cm^−1^) shows a Gaussian-type function as shown in Fig. 4*.* The full width at half maximum (FWHM) gives the waist length of the focal volume (*z* = 30.6 μm), the transverse dimension, *x* and *y* (1.28 μm, 1.28 μm) is determined in view of the numerical aperture of the objective (*N*_*A*_ = 0.75) used for the specific wavelength (*λ*_LASER_ = 785 nm) according to the relation: 1.22 × *λ*_LASER_/*N*_*A*_. The resulting *B*_*υ*_ is 50.1 μm^3^. The number of sampled molecules in the SERS experiment is 2.90 × 10^6^, assuming monolayer formation as reported in literature: thus, the enhancement consists in the fact that populations lower by a factor of around 10^5^ produce comparable signals [22, 27].

It was well evident that, beyond the enhancement effect, the SERS spectrum of pMA was more complex than the spontaneous one. In particular, the SERS peaks at 1011, 1075, 1194, and 1577 cm^−1^ supported a direct correspondence with signals observed in the non-resonant pattern, while the three prominent features at 1146, 1395, and 1444 cm^−1^ had not any counterparts in the spontaneous spectrum. The SERS behavior of pMA is complex and still a matter of debate: according to several authors, the new peaks observed in the SERS pattern were due to the dominance of a specific enhancement mechanism (that can be either electromagnetic or charge-transfer mediated) in a given situation and/or to the orientation of the probe with respect to the substrate surface [28–30]. More recently, it was demonstrated that pMA deposited on a roughened Ag surface reacted to form the dimeric form 4,4′-dimercaptoazobenzene (DMAB) under irradiation with a 632-nm laser line [30]. Quantum chemistry calculations performed on the above compound [31] suggested that the intense signals detected at 1143, 1390, and 1432 cm^−1^ on Ag substrates and at 1142, 1390, and 1440 cm^−1^ on Au substrates [32] and previously interpreted as b_2_ modes of pMA were in fact a_g_ modes of the dimeric species. In view of the close similarity of the SERS pattern obtained in the present study with those reported in refs. [33, 34], we concluded that under the experimental conditions of our SERS experiment, the pMA molecule undergone an extensive conversion to DMAB via a catalytic coupling reaction on the metal surface of the Au-diatom system.

**Fig. 5:**
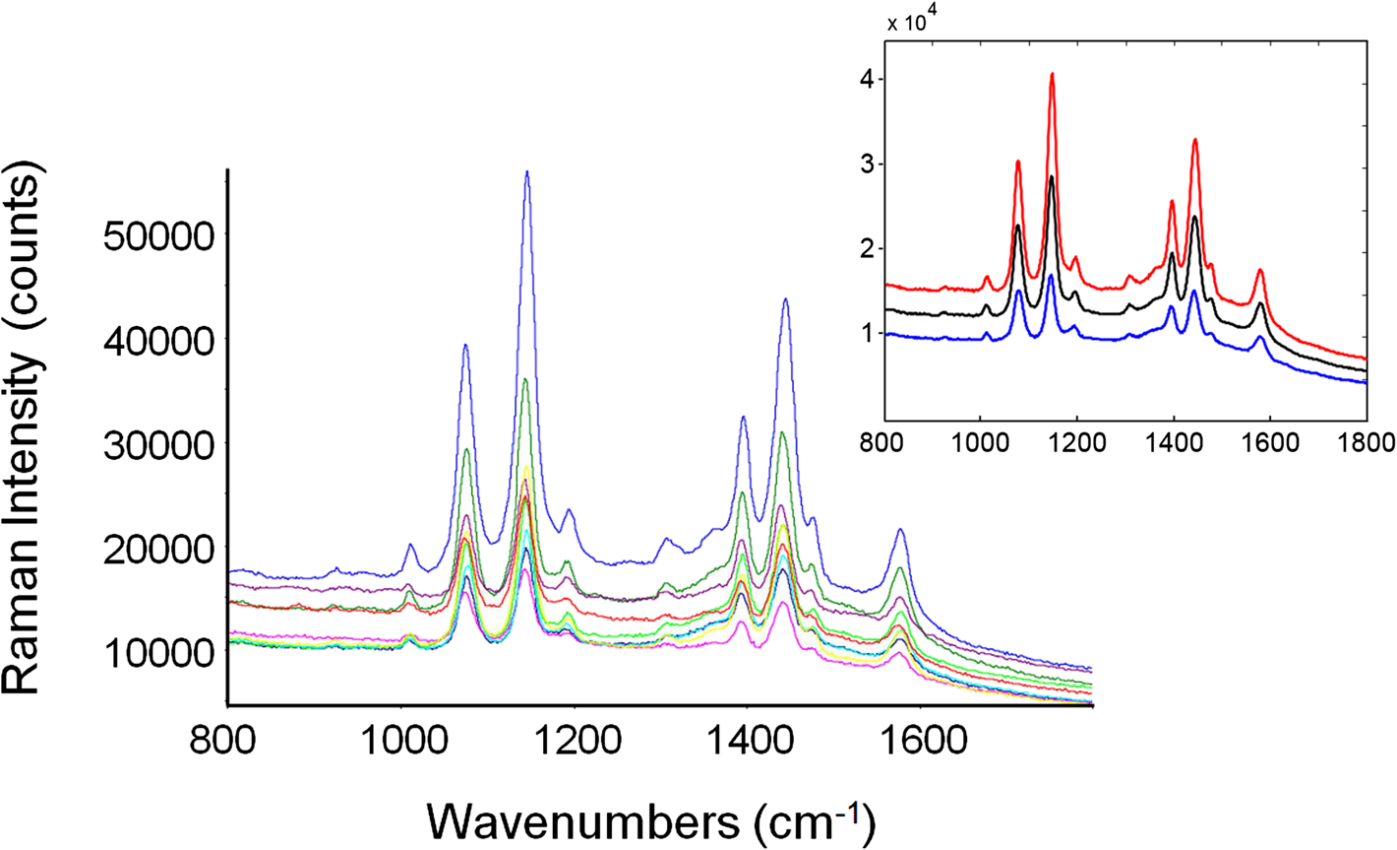
SERS spectra collected on different points of the Au-diatom surface. The *inset* displays the average SERS spectrum (*black trace*) and the average spectrum ± standard deviation (*blue* and *red traces*)

SERS spectra collected on different points of the Au-diatom surface are shown in Fig. 5. The average SERS spectrum and the average spectra ± standard deviations are displayed in the inset of Fig. 5.

The SERS spectra consistently presented the same series of peaks, and the intensity ratio between the main peaks was almost constant. The intensity of single peaks changed from point to point meaning that the Au-SERS response was not completely homogeneous. The spectra enhancement achieved demonstrated that Au-diatom-based substrates could be considered good candidates as SERS substrates, but at the same time, their response was not completely reproducible in terms of intensity. In order to characterize the SERS activity of the Au-diatoms substrate, the enhancement factor (EF) was estimated according to the formula:

$$ \mathrm{E}\mathrm{F}=\frac{{\mathrm{RS}}_{\mathrm{EN}}\kern0.5em  \times {N}_{\mathrm{REF}}}{{\mathrm{RS}}_{\mathrm{REF}}\times {N}_{\mathrm{EN}}}=1.0\times {10}^5 $$

where RS_EN_ and RS_REF_ are the integrated areas of a specific Raman band (at 1092 cm^−1^ in the reference spectrum and at 1075 cm^−1^ in the SERS) for the SERS and the spontaneous Raman spectrum, respectively [33, 34]. *N*_EN_ and *N*_REF_ represent the number of molecules contributing to the SERS and spontaneous Raman scattering, respectively. Assuming total coverage of the sensing surface, the number of molecules contributing to the SERS effect can be estimated as the ratio between the total metal surface illuminated by the laser beam (the transverse dimension) and the geometrical cross section of an individual pMA molecule (0.3 nm^2^ per molecule) [35]. However, the morphological analysis evidenced the presence of ordered lines of nanometric pores, which do not contribute to the available surface area. Thus, a filling factor (FF) needed to be multiplied by the total area in order to account for this effect. The FF is defined as

$$ \mathrm{F}\mathrm{F}=\frac{A_{\mathrm{tot}}-{A}_v}{A_{\mathrm{tot}}} $$

where *A*_tot_ and *A*_*v*_ represent, respectively, the total surface area of the diatom and the total area covered by holes. The average FF, estimated from an image analysis of numerous micrographs as that represented in Fig. 2c, was found to be 0.53.

The gold-diatom substrate was intrinsically heterogeneous on a macroscopic scale with respect to the nanometric dimensions of the sensing surface. This was due to the morphology and size of the diatoms. Therefore, a variable SERS response was expected, as a function of the sampling position in a confocal microscopic collection. In fact, as demonstrated in Fig. 5, the overall pattern was highly reproducible but the peak intensity displays a rather wide variation. From the data of Fig. 5, a maximum EF, EF_max_ of 1.0 × 10^5^ was estimated, while the average EF value is (4.6 ± 2.7) × 10^4^. It should be highlighted that the above values should be considered as conservative estimates since the dimerization reaction decreased the concentration of pMA and, as a consequence, reduced the intensity of the analytical signal. In summary, the present substrate exhibited reasonably high values of EF, which, coupled with the low cost and the relatively simple preparation process, made them an attractive option for realizing highly sensitive SERS-based detectors.

## Conclusions

The efficiency of gold-coated diatoms as SERS substrates was investigated by means of Raman spectroscopy. Gold-coated diatoms were found to be good SERS substrates, enhancing the spontaneous Raman scattering of pMA by a factor of 10^5^. SERS spectra collected in different points on the sensing surface showed a reproducible scattering pattern but a relatively large variability in terms of intensity. This effect was due to the intrinsic heterogeneity at a microscale level of the sensing surface. Despite this limitation, gold-coated diatoms substrate may be considered a low-cost, easy to prepare, and very promising for analytical applications.
